# External Validation of the Ovarian-Adnexal Reporting and Data System (O-RADS) Lexicon and the International Ovarian Tumor Analysis 2-Step Strategy to Stratify Ovarian Tumors Into O-RADS Risk Groups

**DOI:** 10.1001/jamaoncol.2022.5969

**Published:** 2022-12-15

**Authors:** Stefan Timmerman, Lil Valentin, Jolien Ceusters, Antonia C. Testa, Chiara Landolfo, Povilas Sladkevicius, Caroline Van Holsbeke, Ekaterini Domali, Robert Fruscio, Elisabeth Epstein, Dorella Franchi, Marek J. Kudla, Valentina Chiappa, Juan L. Alcazar, Francesco P. G. Leone, Francesca Buonomo, Maria Elisabetta Coccia, Stefano Guerriero, Nandita Deo, Ligita Jokubkiene, Jeroen Kaijser, Giovanni Scambia, Rochelle Andreotti, Dirk Timmerman, Tom Bourne, Ben Van Calster, Wouter Froyman

**Affiliations:** 1 Department of Development and Regeneration, KU Leuven, Leuven, Belgium; 2 Department of Obstetrics and Gynecology, University Hospital Leuven, Leuven, Belgium; 3 Department of Obstetrics and Gynecology, Skåne University Hospital, Malmö, Sweden; 4 Department of Clinical Sciences Malmö, Lund University, Malmö, Sweden; 5 Laboratory of Tumor Immunology and Immunotherapy, Department of Oncology, Leuven Cancer Institute, KU Leuven, Leuven, Belgium; 6 Dipartimento Scienze della Salute della Donna, del Bambino e di Sanità Pubblica, Fondazione Policlinico Universitario A. Gemelli, IRCSS, Rome, Italy; 7 Queen Charlotte’s and Chelsea Hospital, Imperial College, London, United Kingdom; 8 Department of Obstetrics and Gynecology, Ziekenhuis Oost-Limburg, Genk, Belgium; 9 First Department of Obstetrics and Gynecology, Alexandra Hospital, Medical School, National and Kapodistrian University of Athens, Athens, Greece; 10 Clinic of Obstetrics and Gynecology, University of Milan–Bicocca, San Gerardo Hospital, Monza, Italy; 11 Department of Clinical Science and Education, Karolinska Institutet and Department of Obstetrics and Gynecology, Södersjukhuset, Stockholm, Sweden; 12 Preventive Gynecology Unit, Division of Gynecology, European Institute of Oncology IRCCS, Milan, Italy; 13 Department of Perinatology and Oncological Gynecology, Faculty of Medical Sciences, Medical University of Silesia, Katowice, Poland; 14 Department of Gynecologic Oncology, National Cancer Institute of Milan, Milan, Italy; 15 Department of Obstetrics and Gynecology, Clinica Universidad de Navarra, School of Medicine, Pamplona, Spain; 16 Department of Obstetrics and Gynecology, Biomedical and Clinical Sciences Institute L. Sacco, University of Milan, Milan, Italy; 17 Institute for Maternal and Child Health–IRCCS “Burlo Garofolo,” Trieste, Italy; 18 Department of Biomedical, Experimental and Clinical Sciences, University of Florence, Florence, Italy; 19 Department of Obstetrics and Gynecology, University of Cagliari, Policlinico Universitario Duilio Casula, Monserrato, Cagliari, Italy; 20 Department of Obstetrics and Gynaecology, Whipps Cross Hospital, London, United Kingdom; 21 Department of Obstetrics and Gynecology, Ikazia Hospital, Rotterdam, the Netherlands; 22 Department of Radiology and Radiological Sciences, Vanderbilt University Medical Center, Nashville, Tennessee; 23 Department of Obstetrics and Gynecology, Vanderbilt University Medical Center, Nashville, Tennessee; 24 Department of Biomedical Data Sciences, Leiden University Medical Centre, Leiden, the Netherlands

## Abstract

**Importance:**

Correct diagnosis of ovarian cancer results in better prognosis. Adnexal lesions can be stratified into the Ovarian-Adnexal Reporting and Data System (O-RADS) risk of malignancy categories with either the O-RADS lexicon, proposed by the American College of Radiology, or the International Ovarian Tumor Analysis (IOTA) 2-step strategy.

**Objective:**

To investigate the diagnostic performance of the O-RADS lexicon and the IOTA 2-step strategy.

**Design, Setting, and Participants:**

Retrospective external diagnostic validation study based on interim data of IOTA5, a prospective international multicenter cohort study, in 36 oncology referral centers or other types of centers. A total of 8519 consecutive adult patients presenting with an adnexal mass between January 1, 2012, and March 1, 2015, and treated either with surgery or conservatively were included in this diagnostic study. Twenty-five patients were excluded for withdrawal of consent, 2777 were excluded from 19 centers that did not meet predefined data quality criteria, and 812 were excluded because they were already in follow-up at recruitment. The analysis included 4905 patients with a newly detected adnexal mass in 17 centers that met predefined data quality criteria. Data were analyzed from January 31 to March 1, 2022.

**Exposures:**

Stratification into O-RADS categories (malignancy risk <1%, 1% to <10%, 10% to <50%, and ≥50%). For the IOTA 2-step strategy, the stratification is based on the individual risk of malignancy calculated with the IOTA 2-step strategy.

**Main Outcomes and Measures:**

Observed prevalence of malignancy in each O-RADS risk category, as well as sensitivity and specificity. The reference standard was the status of the tumor at inclusion, determined by histology or clinical and ultrasonographic follow-up for 1 year. Multiple imputation was used for uncertain outcomes owing to inconclusive follow-up information.

**Results:**

Median age of the 4905 patients was 48 years (IQR, 36-62 years). Data on race and ethnicity were not collected. A total of 3441 tumors (70%) were benign, 978 (20%) were malignant, and 486 (10%) had uncertain classification. Using the O-RADS lexicon resulted in 1.1% (24 of 2196) observed prevalence of malignancy in O-RADS 2, 4% (34 of 857) in O-RADS 3, 27% (246 of 904) in O-RADS 4, and 78% (732 of 939) in O-RADS 5; the corresponding results for the IOTA 2-step strategy were 0.9% (18 of 1984), 4% (58 of 1304), 30% (206 of 690), and 82% (756 of 927). At the 10% risk threshold (O-RADS 4-5), the O-RADS lexicon had 92% sensitivity (95% CI, 87%-96%) and 80% specificity (95% CI, 74%-85%), and the IOTA 2-step strategy had 91% sensitivity (95% CI, 84%-95%) and 85% specificity (95% CI, 80%-88%).

**Conclusions and Relevance:**

The findings of this external diagnostic validation study suggest that both the O-RADS lexicon and the IOTA 2-step strategy can be used to stratify patients into risk groups. However, the observed malignancy rate in O-RADS 2 was not clearly below 1%.

## Key Points

**Question:**

Can the Ovarian-Adnexal Reporting and Data System (O-RADS) lexicon proposed by the American College of Radiology and the International Ovarian Tumor Analysis (IOTA) 2-step strategy be used to determine the likelihood of malignancy?

**Findings:**

In this diagnostic study of 4905 patients with ovarian tumors, both the O-RADS lexicon and the IOTA 2-step strategy could be used to stratify patients into risk groups. However, the observed malignancy rate in O-RADS 2 was not clearly below 1%.

**Meaning:**

Findings suggest that risk stratification using the O-RADS lexicon or the IOTA 2-step strategy can be used to guide management.

## Teaser

This diagnostic study determines whether the Ovarian-Adnexal Reporting and Data System (O-RADS) lexicon and the International Ovarian Tumor Analysis (IOTA) 2-step strategy can be used to stratify patients into risk groups.

## Introduction

Ovarian cancer is the eighth most common cancer worldwide in women and is the most lethal gynecologic malignancy.^1^ Because patients with ovarian cancer have a better prognosis when treated in tertiary oncology centers than in other centers, correct preoperative diagnosis of adnexal masses should result in optimal management.^2,3,4,5,6^

In 2020, an international multidisciplinary committee, sponsored by the American College of Radiology, published the Ovarian-Adnexal Reporting and Data System (O-RADS).^7^ The system suggests a uniform lexicon to describe ultrasonographic images of adnexal masses, uses this lexicon to stratify masses into different risk groups of malignancy, and suggests management for each risk group.

Another way to classify adnexal masses into O-RADS risk groups than using the O-RADS lexicon is to use a mathematical model to calculate the risk of malignancy (eg, the Assessment of Different Neoplasias in the Adnexa [ADNEX] model developed by the International Ovarian Tumor Analysis [IOTA] group). The ADNEX model is a polynomial logistic regression model that estimates the risk of 5 tumor types: benign, borderline, stage I primary invasive, stage II to IV primary invasive, and secondary metastasis. It is based on ultrasonographic and clinical information and can be used with or without information on serum cancer antigen 125 (CA-125).^8,9^ A third method of risk stratification is to apply the IOTA 2-step strategy (ie, the modified IOTA benign simple descriptors [BDs], which do not require access to a computer, are used first, and if these do not apply, ADNEX is used).^10^ When externally validated with patients treated with surgery or expectantly, this 2-step strategy had good performance.^10^

The ability of the O-RADS lexicon to place patients in the correct O-RADS risk group and the sensitivity and specificity of the O-RADS lexicon regarding malignancy have been validated in studies using retrospective review of images and in 1 small prospective study. In most of these studies, histology was the reference standard.^11,12,13,14,15,16,17,18,19,20,21,22,23,24^ According to the original O-RADS publication, masses with risk of malignancy less than 1% could be managed with follow-up.^7^ Therefore, validation studies should be performed for all patients with an adnexal mass, irrespective of whether they are treated conservatively or with surgery. The aim of this study was to estimate the diagnostic performance of the O-RADS lexicon and the IOTA 2-step strategy when used for both surgically and conservatively managed adnexal masses.

## Methods

### Study Design and Population

This diagnostic study was a retrospective external validation study using the 2-year interim data from IOTA phase 5 (IOTA5), an ongoing international multicenter prospective observational cohort study (ClinicalTrials.gov Identifier: NCT01698632).^9,25^ The IOTA5 study protocol was approved by the ethics committee of the University Hospitals Leuven as the coordinating center and the local ethics committee of each contributing center. The study design has been described elsewhere and is briefly outlined here.^9,25^ The study followed the Standards for Reporting of Diagnostic Accuracy (STARD) reporting guideline.^26^

Patients were eligible for IOTA5 if they were aged 18 years or older at recruitment and had at least 1 adnexal mass (ovarian, paraovarian, or tubal) detected on ultrasonographic examination. Premenopausal patients with a clearly physiologic cyst with largest diameter less than 3 cm were not eligible. The IOTA5 2-year interim analysis data set contains data from patients recruited at 36 centers (oncology or nononcology centers) in 14 countries between January 1, 2012, and March 1, 2015, with follow-up data until June 30, 2017. Patients with an adnexal mass who were already in follow-up in the recruitment center before the start of the study were not used in this analysis.

Written or oral informed consent was obtained from every participant at inclusion. All patients underwent a standardized transvaginal ultrasonographic examination by an IOTA-certified examiner (these examiners had completed a standardized IOTA course and passed an IOTA test on assessment of adnexal masses). Most ultrasonographic examiners were level 2 (experienced) or 3 (advanced) examiners.^27^ By design, the examiners were blinded to the outcome. Results of ultrasonography and clinical information were registered in accordance with research protocol.^9,24^ IOTA terminology was used to describe the ultrasonographic findings,^28^ and a set of predefined ultrasonographic variables was collected for each patient.^9,25^ In addition, the ultrasonographic examiner’s diagnosis (benign, borderline, or malignant; specific diagnosis from a drop-down list), based on pattern recognition (ie, based on knowledge of the typical ultrasonographic appearance of different types of adnexal pathology^29^), was recorded together with the confidence with which the diagnosis was made (certainly benign, probably benign, uncertain, probably malignant, or certainly malignant). The ultrasonographic examiner suggested management based on clinical findings and the ultrasonographic diagnosis. The treating clinician decided on the final management together with the patient. Conservative management included ultrasonography and clinical follow-up at intervals of 3 months, 6 months, and then every 12 months. At follow-up visits, clinical data (including symptoms) were collected, and an ultrasonographic examination was performed in the same manner as at the inclusion scan. Some patients initially treated conservatively underwent surgery after 1 or more follow-up visits (eg, because of suspicion of malignancy, symptoms, patient anxiety). In some patients, the mass resolved spontaneously during follow-up. In case of multiple masses, the mass with the ultrasonographic features most suggestive of malignancy was defined as dominant and was used in our statistical analysis.

Patients who underwent surgery were treated according to local protocols, with histologic examination of surgically removed masses. Central pathology review was not performed because, in a previous study, we did not observe important differences in diagnoses between local and central pathology reports.^30^ Malignant tumors were classified according to International Federation of Gynecology and Obstetrics recommendations.^31^

### Data Collection and Cleaning

Patient data were collected on a secure electronic platform developed for the study (IOTA5 Study Screen; astraia software, version 2.0_58). Patients were pseudonymized on inclusion with a unique identifier, ensuring encryption of all data communication. A team of biostatisticians and ultrasonographic examiners cleaned the data, which included correcting inconsistencies and retrieving missing information. Before analyzing the data, we defined the criteria for a study center to be included in our analysis: recruitment of at least 50 patients, consecutive recruitment, and good-quality follow-up data (ie, a recorded study outcome or last follow-up visit ≥10 months after inclusion) for at least 70% of the recruited patients.^9^

### Reference Standard

The reference standard describes the nature of the adnexal mass as benign or malignant at inclusion (borderline tumors were classified as malignant). It is based on histology for masses removed by surgery. Pathologists were blinded to ultrasonographic variables, risk predictions, and O-RADS groups but might have received information on the diagnosis suspected by the ultrasonographic examiner. If surgery was not performed, the reference standard was based on clinical history and status and the ultrasonographic examiner’s diagnosis and diagnostic confidence at inclusion and during follow-up until 12 months (±2 months). Table 1 shows how we determined tumor outcome.^9^

**Table 1.** Definition of Tumor Outcome Based on Histology or Clinical Information

Outcome and scenario	Tumors, No. (%) (N = 4905)
Benign	
B1: Surgery, benign histology	2065 (42)
B1.1: Surgery within 120 d without FU visit, benign histology	1544 (31)
B1.2: Surgery after 120 d or after ≥1 FU visit, benign histology	521 (11)
B2: No surgery, no spontaneous resolution, last visit ≥10 mo, SA at every visit up to 10-14 mo probably benign or certainly benign	911 (19)
B3: Spontaneous resolution	465 (9)
Malignant	
M1: Surgery within 120 d, malignant histology	956 (19)
M2: Surgery after 120 d, malignant histology, SA at every visit up to surgery probably borderline or probably malignant or certainly borderline or certainly malignant	18 (0.4)
M3: No surgery, no spontaneous resolution, last visit ≥10 mo, SA at every visit up to 10-14 mo probably borderline or probably malignant or certainly borderline or certainly malignant	4 (0.1)^a^
Uncertain	
U1: Surgery after 120 d, malignant histology, SA not probably borderline or probably malignant or certainly borderline or certainly malignant at every visit up to surgery	19 (0.4)
U2: No surgery, no spontaneous resolution, last visit ≥10 mo, SA uncertain or inconsistent across visits up to 10-14 mo	35 (0.7)
U3: No surgery, no spontaneous resolution, last FU visit was before 10 mo (owing to death, withdrawal from study, or lost to FU)	123 (3)
U4: No information after the inclusion visit	309 (6)

Abbreviations: FU, follow-up; SA, subjective assessment.

^a^ For these tumors, type of cancer could not be determined. Type of cancer was treated as a missing value and imputed.

### O-RADS Risk Stratification

O-RADS 1 indicates a normal ovary, which is not applicable here. O-RADS 2 indicates an almost certainly benign tumor (malignancy risk <1%), O-RADS 3 indicates low risk (malignancy risk 1% to <10%), O-RADS 4 indicates intermediate risk (malignancy risk 10% to <50%), and O-RADS 5 indicates high risk (malignancy risk ≥50%).^7^ Table 2 shows how we derived the O-RADS lexicon from the 2-year interim IOTA5 database.

**Table 2.** IOTA Terms and Variables Used to Translate the IOTA Terminology Into the O-RADS Lexicon^a^

O-RADS lexicon	IOTA terms and variables corresponding to the O-RADS lexicon
**O-RADS 2**	
2a: Simple cyst	Locularity = unilocular; irregular = no; echogenicity = anechoic; lesion largest diameter <10 cm
2a1: ≤3 cm	Lesion largest diameter ≤3 cm
2a2: >3-5 cm	Lesion largest diameter >3 to ≤5 cm
2a3: >5 but <10 cm	Lesion largest diameter >5 to <10 cm
2b: Classic benign lesions	
2b1: Typical hemorrhagic cyst <10 cm	Subjective assessment: hemorrhagic cyst, probably or certainly benign; lesion largest diameter <10 cm
2b2: Typical dermoid cyst <10 cm	Subjective assessment: dermoid cyst, probably or certainly benign; lesion largest diameter <10 cm
2b3: Typical endometrioma <10 cm	Subjective assessment: endometrioma, probably or certainly benign; lesion largest diameter <10 cm
Peritoneal inclusion cyst	Subjective assessment: peritoneal inclusion cyst, probably or certainly benign
Hydrosalpinx	Subjective assessment: hydrosalpinx, probably or certainly benign
2c: Nonsimple unilocular cyst, smooth inner margin	Locularity = unilocular; irregular = no; lesion largest diameter <10 cm
2c1: ≤3 cm	Lesion largest diameter ≤3 cm
2c2: >3 but <10 cm	Lesion largest diameter >3 to <10 cm
**O-RADS 3**	
3a: Unilocular cyst ≥10 cm (simple or nonsimple)	Locularity = unilocular; irregular = no; lesion largest diameter ≥10 cm
3b: Typical dermoid cysts, endometrioma, hemorrhagic cysts ≥10 cm	
3b1: Typical hemorrhagic cyst ≥10 cm	Subjective assessment: hemorrhagic cyst, probably or certainly benign; lesion largest diameter ≥10 cm
3b2: Typical dermoid cyst ≥10 cm	Subjective assessment: dermoid cyst, probably or certainly benign; lesion largest diameter ≥10 cm
3b3: Typical endometrioma ≥10 cm	Subjective assessment: endometrioma, probably or certainly benign; lesion largest diameter ≥10 cm
3c: Unilocular cyst, any size with irregular wall <3 mm height	Locularity = unilocular; irregular = yes
3d: Multilocular cyst <10 cm, smooth inner wall, color score = 1-3	Locularity = multilocular; irregular = no; color score <4; lesion largest diameter <10 cm
3e: Solid smooth, any size, color score = 1	Locularity = solid; irregular = no; color score = 1
**O-RADS 4**	
4a: Multilocular cyst, no solid component	
4a1: ≥10 cm, smooth inner wall, color score = 1-3	Locularity = multilocular; irregular = no; color score <4; lesion largest diameter ≥10 cm
4a2: Any size, smooth inner wall, color score = 4	Locularity = multilocular; irregular = no; color score = 4
4a3: Any size; irregular inner wall, irregular septation, or both; color score = any	Locularity = multilocular; irregular = yes
4b: Unilocular cyst with solid component, any size, 0-3 papillary projections, color score = any	Locularity = unilocular, solid; No. of papillary projections <4
4c: Multilocular cyst with solid component, any size, color score = 1-2	Locularity = multilocular, solid; color score <3
4d: Solid, smooth, any size, color score = 2-3	Locularity = solid; irregular = no; color score = 2 or 3
**O-RADS 5**	
5a: Unilocular cyst, any size, ≥4 papillary projections, color score = any	Locularity = unilocular, solid; No. of papillary projections ≥4
5b: Multilocular cyst with solid component, any size, color score = 3-4	Locularity = multilocular, solid; color score >2
5c: Solid smooth, any size, color score = 4	Locularity = solid; irregular = no; color score = 4
5d: Solid irregular, any size, color score = any	Locularity = solid; irregular = yes
5e: Ascites, peritoneal nodules, or both	Ascites = yes or metastases = yes

Abbreviations: IOTA, International Ovarian Tumor Analysis; O-RADS, Ovarian-Adnexal Reporting and Data System.

^a^ Color score is based on the IOTA terms, with a score of 1 when no blood flow can be found in the lesion, 2 when only minimal flow can be detected, 3 when moderate flow is present, and 4 when the adnexal mass appears highly vascular with marked blood flow.^28^ There is no unequivocal O-RADS definition of typical hemorrhagic cyst, typical dermoid cyst, typical endometrioma, typical hydrosalpinx, or typical peritoneal inclusion cyst. Therefore, the ultrasonographic examiner’s diagnosis based on subjective assessment was used to assign masses to these O-RADS subcategories. The IOTA term *metastasis* was used as a surrogate for the O-RADS lexicon term *peritoneal nodule*. For the assignment of tumors into the O-RADS categories, we checked the features in top-to-bottom order, with the following exceptions: (1) O-RADS 3b was evaluated before O-RADS 3a (first check whether size is ≥10 cm and then the presence of a typical lesion; if ≥10 cm but a typical lesion is not present and the cyst is unilocular smooth [ie, irregular absent], it is classified as 3a); and (2) Andreotti et al^7^ stated that, when ascites is present in combination with a tumor that qualifies for O-RADS 2, other etiologies for the presence of ascites should be considered. In this work, when ascites was found in a patient with an O-RADS 2 tumor, the tumor was assigned to O-RADS 2. In all other cases, the presence of ascites led to assignment into O-RADS 5 (5e). The presence of metastasis always implied assignment into O-RADS 5 (5e).

### Two-Step Strategy

The modified IOTA BDs almost always indicate a benign tumor according to studies of patients who underwent surgery.^32,33^ This corresponds to O-RADS 2 (risk of malignancy <1%). The BDs are BD1 (unilocular cyst, ground-glass echogenicity, largest diameter <10 cm, and premenopausal patient), BD2 (unilocular cyst, mixed echogenicity, acoustic shadows, largest diameter <10 cm, and premenopausal patient), BD3 (unilocular cyst, anechoic cyst fluid, smooth internal walls, and largest diameter <10 cm), and BD4 (remaining unilocular cysts with smooth internal walls and largest diameter <10 cm).

If the BDs do not apply, ADNEX is used, which calculates the risk of 5 tumor types: benign, borderline, stage I invasive cancer, stage II to IV invasive cancer, and secondary metastasis.^9^ The risk of malignancy is obtained by adding the risks of the 4 malignant tumor types. In this study, we used ADNEX without serum CA-125 because CA-125 results are usually not known when a patient presents with an adnexal mass. The risks of malignancy generated by ADNEX were divided into 4 categories corresponding to the malignancy probabilities of O-RADS 2 to 5.

### Statistical Analysis

We used multiple imputation to deal with uncertain outcomes (U1-U4 in Table 1). We generated 100 imputations and refer to previous work for details.^9^ Our results are based on the multiply imputed values for the outcome.

The percentage of patients was calculated, as well as the histologic outcome (benign, borderline, stage I invasive, stage II-IV invasive, and secondary metastatic) in each O-RADS risk group (pooled analysis). Sensitivity, specificity, positive predictive value, and negative predictive value were calculated for the O-RADS lexicon and the IOTA 2-step strategy at 3 O-RADS risk thresholds: 1% (O-RADS ≥3 vs O-RADS 2), 10% (O-RADS 4-5 vs O-RADS 2-3), and 50% (O-RADS 5 vs O-RADS 2-4) (meta-analysis). To deal with multiply imputed data, logit-transformed values for sensitivity and specificity were combined using Rubin rules to obtain center-specific results. These center-specific results (logit transformed) were combined with bivariate random-effects meta-analysis to calculate the overall sensitivity and specificity.^34^ Center-specific positive and negative predictive values (logit transformed) and their variance were computed and combined using Rubin rules to obtain a final center-specific estimate. These estimates were used in a bivariate random-effects model to calculate the final estimate.^35^

We performed predetermined subgroup analyses depending on menopausal status and type of center (oncology center vs other). We also performed 2 post hoc subgroup analyses defined by actual management: patients who underwent surgery within 120 days of the inclusion scan without any follow-up scan, and patients with at least 1 follow-up scan.

Some ultrasonographic features are not included in the original O-RADS classification. At the request of the members of the American College of Radiology O-RADS committee, we calculated the observed prevalence of malignancy when taking into account the echogenicity of cyst fluid (anechoic vs other), number of cyst locules in multilocular cysts (2 cyst locules vs >2), and presence of shadowing in smooth solid tumors.

The statistical analysis was performed with R, version 4.1.0 (R Foundation for Statistical Computing). We used the mice package for multiple imputation and the metafor package (rma.mv function) for the meta-analysis of diagnostic performance. Data were analyzed from January 31, 2022, to March 1, 2022.

## Results

The Figure shows patient flow, with an initial consecutive cohort of 8519 patients from 36 centers. Our statistical analysis included the data of all 4905 patients in the 17 centers that fulfilled our predetermined data quality criteria^9^: 3441 (70%) tumors were benign, 978 (20%) were malignant, and for 486 tumors (10%), the outcome was uncertain and imputed. Patient and tumor characteristics are presented in eTable 1 in the Supplement. Median age was 48 years (IQR, 36-62 years). Data on race and ethnicity were not collected. eTable 2 in the Supplement shows how final tumor outcome was determined for each O-RADS risk group.

**Figure.**
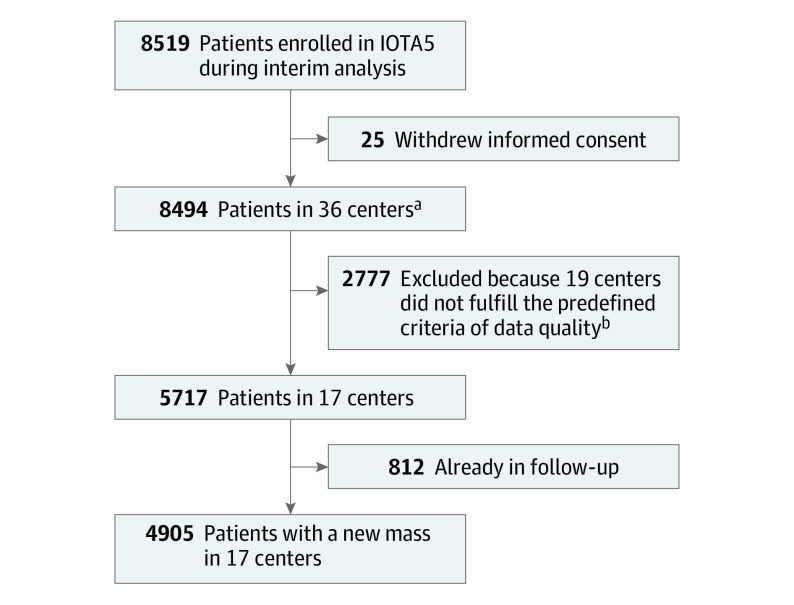
Study Flowchart IOTA5 indicates International Ovarian Tumor Analysis phase 5 study. ^a^From the 8494 patients in 36 centers, the tumor outcome was benign in 5720 (67%), malignant in 1342 (16%), and uncertain in 1432 (17%). ^b^Predefined criteria for including centers: at least 50 patients recruited, consecutive recruitment, and good follow-up information for at least 70% of recruited patients. Good quality of follow-up data was defined as a recorded study outcome (spontaneous resolution, surgery with histology, or death) or last follow-up scan 10 months or more after the inclusion scan.

Using the O-RADS lexicon for risk stratification resulted in 1.1% (24 of 2196) observed prevalence of malignancy in O-RADS 2, 4% (34 of 857) in O-RADS 3, 27% (246 of 904) in O-RADS 4, and 78% (732 of 939) in O-RADS 5; the corresponding results for the IOTA 2-step strategy were 0.9% (18 of 1984), 4% (58 of 1304), 30% (206 of 690), and 82% (756 of 927) (Table 3; eTable 3 in the Supplement). With the exception of O-RADS 2 when the O-RADS lexicon was used, the observed proportion of malignant tumors per O-RADS group fell within the targeted risk range. eTable 4 in the Supplement shows the observed number and percentage of malignant tumors in each of the 33 O-RADS subgroup categories. The observed malignancy rate was less than 1% for simple cysts, classic hemorrhagic cysts, dermoid cysts, and endometriomas less than 10 cm, and for nonsimple unilocular cysts with smooth inner margin less than or equal to 3 cm.

**Table 3.** Observed Prevalence of Malignancy per O-RADS Group^a^

O-RADS	No. (%)	Malignant, % (95% CI)^b^
O-RADS lexicon		
O-RADS		
2 (<1%)	2196 (45)	1.1 (0.7-1.6)
3 (1 to <10%)	857 (17)	4 (3-6)
4 (10 to <50%)	904 (18)	27 (24-30)
5 (≥50%)	939 (19)	78 (75-81)
Unclassified^c^	9 (0.2)	14 (2-56)
IOTA 2-step strategy		
<1%	1984 (40)	0.9 (0.6-1.5)
1 to <10%	1304 (27)	4 (3-6)
10 to <50%	690 (14)	30 (26-33)
≥50%	927 (19)	82 (79-84)

Abbreviations: IOTA, International Ovarian Tumor Analysis; O-RADS, the Ovarian-Adnexal Reporting and Data System.

^a^ When the O-RADS lexicon and the IOTA 2-step strategy were used to estimate the risk of malignancy (N = 4905; pooled analysis).

^b^ Percentages were rounded except those below 2%.

^c^ It was not possible to classify 9 patients with the O-RADS lexicon because the tumor type was listed as “unclassifiable” in the IOTA database, and there was no ascites or metastasis.

Sensitivity, specificity, positive predictive value, and negative predictive value regarding malignancy when the O-RADS lexicon and the IOTA 2-step strategy were used are shown in Table 4. At the 10% risk threshold (O-RADS 4-5 vs O-RADS 2-3), the O-RADS lexicon had 92% sensitivity (95% CI, 87%-96%) and 80% specificity (95% CI, 74%-85%), and the 2-step strategy had 91% sensitivity (95% CI, 84%-95%) and 85% specificity (95% CI, 80%-88%).

**Table 4.** Sensitivity, Specificity, and Positive and Negative Predictive Value Regarding Malignancy^a^

Cutoff for malignancy for O-RADS lexicon and for percentage risk of malignancy	Sensitivity (95% CI)		Specificity (95% CI)		Predictive value (95% CI)			
					Positive		Negative	
	O-RADS lexicon	IOTA 2-step strategy	O-RADS lexicon	IOTA 2-step strategy	O-RADS lexicon	IOTA 2-step strategy	O-RADS lexicon	IOTA 2-step strategy
O-RADS								
3 (1%)	0.97 (0.94-0.98)	0.97 (0.94-0.98)	0.58 (0.50-0.65)	0.52 (0.46-0.59)	0.37 (0.28-0.48)	0.34 (0.26-0.44)	0.99 (0.98-0.99)	0.99 (0.98-0.99)
4 (10%)	0.92 (0.87-0.96)	0.91 (0.84-0.95)	0.80 (0.74-0.85)	0.85 (0.80-0.88)	0.55 (0.44-0.65)	0.60 (0.51-0.68)	0.98 (0.97-0.98)	0.98 (0.97-0.98)
5 (50%)	0.66 (0.58-0.73)	0.67 (0.56-0.76)	0.96 (0.94-0.98)	0.96 (0.94-0.98)	0.80 (0.71-0.87)	0.81 (0.74-0.87)	0.92 (0.89-0.94)	0.92 (0.90-0.94)

Abbreviations: IOTA, International Ovarian Tumor Analysis; O-RADS, Ovarian-Adnexal Reporting and Data System.

^a^ When the O-RADS lexicon and the IOTA 2-step strategy were used (meta-analysis). Value at cutoff or higher classifies the mass as malignant.

The results of our subgroup analyses are shown in eTable 5 and eTable 6 in the Supplement. For both the O-RADS lexicon and the IOTA 2-step strategy, the observed proportion of malignant tumors in O-RADS 2 was greater than 1% in postmenopausal patients (1.8%) and in oncology centers (1.4% and 1.2%), whereas it was less than 1% in premenopausal patients (0.8% and 0.6%) and in nononcology centers (0.7% and 0.6%). The observed proportion of malignant tumors in the other O-RADS groups fell within the targeted risk range irrespective of menopausal status and type of center (eTable 2 in the Supplement). Sensitivity was higher and specificity lower in postmenopausal than premenopausal patients and in oncology centers than in other centers. In patients who underwent immediate surgery, the observed malignancy rate was higher in all O-RADS risk groups than in those with at least 1 follow-up scan, and sensitivity was higher and specificity lower.

eTable 7 in the Supplement shows the observed prevalence of malignancy in O-RADS groups 3a, 3d, 3e, 4d, and 5c when number of cyst locules (O-RADS 3d), echogenicity of cyst fluid (O-RADS 3a and 3d), and shadowing (O-RADS 3e, 4d, and 5c) were taken into account. For the 183 bilocular cysts in O-RADS subcategory 3d, the observed prevalence of malignancy was 0.7%. In O-RADS subcategories 3e, 4d, and 5c (smooth solid tumors), the observed prevalence of malignancy was substantially lower if acoustic shadows were present.

## Discussion

Our results showed that both the O-RADS lexicon and the IOTA 2-step strategy performed well to stratify patients into O-RADS groups 3 to 5. For O-RADS 2, the target proportion of malignant tumors is less than 1%, but the observed proportions were not clearly below 1%. The large amount of multicentric data from patients treated conservatively or surgically suggests generalizability of these results.

We reported the observed rate of malignancy in the O-RADS groups separately for premenopausal and postmenopausal patients and, to our knowledge, for the first time, validated O-RADS separately in oncology centers and other types of centers. The higher sensitivity and lower specificity and the higher observed prevalence of malignancy in lesions classified as O-RADS 2 in postmenopausal patients and in patients examined in oncology centers are likely to be explained by differences in histologic diagnoses (“case mix”) between premenopausal and postmenopausal patients and between oncology and other centers. The difference in performance between patients who underwent surgery and those cared for with follow-up also illustrates the association between study population characteristics and results. Patients who undergo surgery constitute a highly select population in which the proportion of malignant tumors is higher than in a total population of patients with an adnexal mass. Patients treated expectantly with follow-up constitute another select population, in which the malignancy rate is very low. We believe that the best estimate of performance of the O-RADS lexicon and the IOTA 2-step strategy is obtained by including all patients with an adnexal mass irrespective of how they were treated. Our results also show that clinicians were good at selecting patients for conservative management by using clinical information and pattern recognition to interpret ultrasonographic images.

Use of the O-RADS lexicon to stratify patients into O-RADS risk groups has been validated retrospectively in 13 other studies^11,12,13,14,15,16,17,18,19,20,21,22,24^ and prospectively in 1 study^23^ (search strategy and study details shown in the eAppendix and eTable 8 in the Supplement). The prospective study was small (50 patients) and included only tumors in O-RADS 3 to 5.^23^ All retrospective studies used review of saved ultrasonographic images with or without supplementary review of ultrasonographic reports. In 9 retrospective studies, the reference standard was histology,^12,13,14,15,17,18,19,20,22^ in 3 it was histology or results of follow-up,^11,16,24^ and in 1 it was the agreed diagnosis of 2 radiologists according to the ultrasonographic images.^21^ The reported observed malignancy rates per O-RADS group and the sensitivities and specificities when O-RADS 4 to 5 was used (≥10% malignancy risk) to indicate malignancy are variable. The point estimates of observed prevalence of malignancy in O-RADS 2 ranges from 0% to 5%, with 4 centers reporting it to be greater than 1%^12,15,18,22^; that in O-RADS 3 ranges from 0% to 19%, with 4 centers reporting it to be greater than 10%^15,18,22,23^; that in O-RADS 4 ranges from 21% to 79%, with 4 centers reporting it to be greater than 50%^12,15,18,23^; and that in O-RADS 5 ranges from 66% to 95%. The divergent results are likely to be explained by differences in study population characteristics (eg, types of tumors included sample size, study design, interpretation of the O-RADS lexicon) and by a varying proportion of images that were not fully representative of the tumor. Three studies compared sensitivity and specificity at the 10% risk cutoff (O-RADS 4-5) between use of the O-RADS lexicon and use of ADNEX to estimate the risk of malignancy, again with divergent results.^12,13,17^ To our knowledge, no study reported the observed prevalence of malignancy when risk stratification was done with ADNEX or the IOTA 2-step strategy.

Risk categorization may help when patients are selected for treatment. To facilitate the use of O-RADS and ADNEX, web applications and mobile applications are available,^36,37^ and ADNEX is incorporated in some ultrasound machines.^10^ The advantage of the IOTA 2-step strategy over the O-RADS lexicon is that it provides an individual risk estimate of malignancy and estimates the likelihood of different tumor types (benign, borderline, stage I invasive malignancy, stage II to IV invasive malignancy, and secondary metastasis).^8,10^ However, neither the O-RADS lexicon nor the 2-step strategy confidently identified tumors with malignancy risk less than 1%. When the 2-step strategy was used to place tumors in O-RADS 2, the upper 95% confidence limit for the observed prevalence of malignancy exceeded 1%, and when the O-RADS lexicon was used, the point estimate exceeded 1%. The performance of the O-RADS lexicon might be improved by taking into account the number of cyst locules, echogenicity of cyst fluid, and shadowing, subdividing the O-RADS subcategories further. However, increasing the number of subgroups will make use of the O-RADS lexicon more difficult.

### Limitations

Our study has limitations. First, although ultrasonographic information was collected prospectively, the O-RADS lexicon was applied retrospectively. A prospective study would yield results more similar to expected performance in clinical practice. Second, several centers were excluded because of few recruited patients, nonconsecutive recruitment, or insufficient data quality. Third, our reference standard is based on different methods: histology or results of clinical and ultrasonographic follow-up (differential verification).^38^ In some cases, the outcome was unclear because of insufficient or inconsistent information (partial verification). We dealt with this by using multiple imputation.^39^

## Conclusions

The findings of this external diagnostic validation study suggest that both the O-RADS lexicon and the IOTA 2-step strategy can be used to stratify patients into risk groups. However, the observed malignancy rate in O-RADS 2 was not clearly less than 1%. The advantage of the 2-step strategy is that it provides an individual risk estimate, as well as risk estimates of 4 types of malignancy. Prospective validation of the 2 approaches to risk stratification is needed.

## Notes

Supplement. **eTable 1.** Patient and Tumor Characteristics **eTable 2.** Outcome Determination by O-RADS Category **eTable 3.** Observed Number and Percentage of Different Tumor Types in Each Ovarian-Adnexal Reporting and Data System (O-RADS) Risk Group When Using the O-RADS Lexicon and When Using the International Ovarian Tumor Analysis (IOTA) 2-Step Strategy to Estimate the Malignancy Risk (Pooled Analysis) **eTable 4.** Observed Number and Percentage of Malignant Tumors in Each Ovarian-Adnexal Reporting and Data System (O-RADS) Subgroup Category (Pooled Analysis) **eTable 5.** Observed Prevalence of Malignancy per O-RADS Group Depending on Menopausal Status, Type of Center, or the Actual Management When Using the Ovarian-Adnexal Reporting and Data System (O-RADS) Lexicon and the International Ovarian Tumor Analysis (IOTA) 2-Step Strategy to Estimate the Risk of Malignancy (Pooled Analysis) **eTable 6.** Sensitivity and Specificity With Regard to Malignancy of the Ovarian-Adnexal Reporting and Data System (O-RADS) Lexicon and International Ovarian Tumor Analysis (IOTA) 2-Step Strategy Depending on Menopausal Status, Type of Center, and Actual Management (Meta-analysis) **eTable 7.** Observed Prevalence of Malignancy in Ovarian-Adnexal Reporting and Data System (O-RADS) Groups 3a, 3d, 3e, 4d, and 5c When Number of Cyst Locules (O-RADS 3d), Echogenicity of Cyst Fluid (O-RADS 3a, 3d), and Shadowing (O-RADS 3e, 4d, and 5c) Are Taken Into Account (Pooled Analysis) **eAppendix.** Search Strategy to Find Publications on Validation of Ovarian Adnexal Reporting and Data System (O-RADS) **eTable 8.** Summary of Studies Validating the Ovarian Adnexal Reporting and Data System (O-RADS)
eReferences.
