# Supplementary material for: External Validation of the Ovarian-Adnexal Reporting and Data System (O-RADS) Lexicon and the International Ovarian Tumor Analysis 2-Step Strategy to Stratify Ovarian Tumors Into O-RADS Risk Groups
Source: JAMA Oncol. 2022 Dec 15;9(2):225–33. doi: 10.1001/jamaoncol.2022.5969 (PMC9856950; doi:10.1001/jamaoncol.2022.5969)
Supplement: Supplement. — eTable 1. Patient and Tumor Characteristics eTable 2. Outcome Determination by O-RADS Category eTable 3. Observed Number and Percentage of Different Tumor Types in Each Ovarian-Adnexal Reporting and Data System (O-RADS) Risk Group When Using the O-RADS Lexicon and When Using the International Ovarian Tumor Analysis (IOTA) 2-Step Strategy to Estimate the Malignancy Risk (Pooled Analysis) eTable 4. Observed Number and Percentage of Malignant Tumors in Each Ovarian-Adnexal Reporting and Data System (O-RADS) Subgroup Category (Pooled Analysis) eTable 5. Observed Prevalence of Malignancy per O-RADS Group Depending on Menopausal Status, Type of Center, or the Actual Management When Using the Ovarian-Adnexal Reporting and Data System (O-RADS) Lexicon and the International Ovarian Tumor Analysis (IOTA) 2-Step Strategy to Estimate the Risk of Malignancy (Pooled Analysis) eTable 6. Sensitivity and Specificity With Regard to Malignancy of the Ovarian-Adnexal Reporting and Data System (O-RADS) Lexicon and International Ovarian Tumor Analysis (IOTA) 2-Step Strategy Depending on Menopausal Status, Type of Center, and Actual Management (Meta-analysis) eTable 7. Observed Prevalence of Malignancy in Ovarian-Adnexal Reporting and Data System (O-RADS) Groups 3a, 3d, 3e, 4d, and 5c When Number of Cyst Locules (O-RADS 3d), Echogenicity of Cyst Fluid (O-RADS 3a, 3d), and Shadowing (O-RADS 3e, 4d, and 5c) Are Taken Into Account (Pooled Analysis) eAppendix. Search Strategy to Find Publications on Validation of Ovarian Adnexal Reporting and Data System (O-RADS) eTable 8. Summary of Studies Validating the Ovarian Adnexal Reporting and Data System (O-RADS) eReferences. [file jamaoncol-e225969-s001.pdf]

Supplementary Online Content

Timmerman S, Valentin L, Ceusters J, et al. External validation of the Ovarian-Adnexal Reporting and Data System (O-RADS) lexicon and the International Ovarian Tumor Analysis 2-step strategy to stratify ovarian tumors into O-RADS risk groups. *JAMA Oncol*. Published online December 15, 2022. doi:10.1001/jamaoncol.2022.5969

eTable 1. Patient and Tumor Characteristics

eTable 2. Outcome Determination by O-RADS Category

eTable 3. Observed Number and Percentage of Different Tumor Types in Each Ovarian-Adnexal Reporting and Data System (O-RADS) Risk Group When Using the O-RADS Lexicon and When Using the International Ovarian Tumor Analysis (IOTA) 2-Step Strategy to Estimate the Malignancy Risk (Pooled Analysis)

eTable 4. Observed Number and Percentage of Malignant Tumors in Each Ovarian-Adnexal Reporting and Data System (O-RADS) Subgroup Category (Pooled Analysis)

eTable 5. Observed Prevalence of Malignancy per O-RADS Group Depending on Menopausal Status, Type of Center, or the Actual Management When Using the Ovarian-Adnexal Reporting and Data System (O-RADS) Lexicon and the International Ovarian Tumor Analysis (IOTA) 2-Step Strategy to Estimate the Risk of Malignancy (Pooled Analysis)

eTable 6. Sensitivity and Specificity With Regard to Malignancy of the Ovarian-Adnexal Reporting and Data System (O-RADS) Lexicon and International Ovarian Tumor Analysis (IOTA) 2-Step Strategy Depending on Menopausal Status, Type of Center, and Actual Management (Meta-analysis)

eTable 7. Observed Prevalence of Malignancy in Ovarian-Adnexal Reporting and Data System (O-RADS) Groups 3a, 3d, 3e, 4d, and 5c When Number of Cyst Locules (O-RADS 3d), Echogenicity of Cyst Fluid (O-RADS 3a, 3d), and Shadowing (O-RADS 3e, 4d, and 5c) Are Taken Into Account (Pooled Analysis)

eAppendix. Search Strategy to Find Publications on Validation of Ovarian Adnexal Reporting and Data System (O-RADS)

eTable 8. Summary of Studies Validating the Ovarian Adnexal Reporting and Data System (O-RADS)

eReferences.

© 2022 Timmerman S et al. *JAMA Oncology*.

This supplementary material has been provided by the authors to give readers additional information about their work.

© 2022 Timmerman S et al. *JAMA Oncology*.

eTable 1. Patient and Tumor Characteristics

Variable	Median (IQR), or n (%)
Patient age at recruitment (years)	
Median and interquartile range	48 (IQR 36 – 62)
Range	18 – 98
Postmenopausal*	2151 (44%)
Largest diameter of lesion (mm)	
Median and interquartile range	55 (38 – 83)
Range	7 – 751
Tumor type using IOTA terminology	
Unilocular	2140 (44%)
Unilocular-solid	396 (8%)
Multilocular	1011 (21%)
Multilocular-solid	649 (13%)
Solid	689 (14%)
Not possible to classify	20 (0.4 %)
Largest diameter of largest solid component†	
Median and interquartile range	41 (19 - 68)
Range	3 - 751
Number of papillary projections	
None	4335 (88%)
1	282 (6%)
2	85 (2%)
3	49 (1%)
More than 3	154 (3%)
More than 10 cyst locules	368 (8%)
Irregular internal cyst walls	1502 (31%)
Echogenicity of cyst fluid	
Anechoic	1852 (38%)
Low level	778 (16%)
Ground glass	793 (16%)
Mixed	680 (14%)

© 2022 Timmerman S et al. *JAMA Oncology*.

Hemorrhagic	113 (2%)
Not applicable	689 (14%)
Acoustic shadows	754 (15%)
Color score, n (%)	
1: no blood flow	2031 (41%)
2: minimal blood flow	1336 (27%)
3: moderate blood flow	1099 (22%)
4: very strong flow	439 (9%)
Ascites	285 (6%)
Presence of metastasis	338 (7%)
Ultrasound examiner’s subjective impression	
Certainly benign	2488 (51%)
Probably benign	1066 (22%)
Uncertain	367 (7%)
Probably malignant	392 (8%)
Certainly malignant	592 (12%)
Ultrasound examiner’s presumed diagnosis	
Abcess/salpingitis/PID	88 (2%)
Benign rare tumour	19 (0.4%)
Borderline malignant tumour	218 (4%)
Endometrioma	742 (15%)
Fibroma/fibrothecoma	216 (4%)
Functional cyst	184 (4%)
Hydrosalpinx	156 (3%)
Inclusion cyst/peritoneal cyst	36 (1%)
Malignant rare tumour	97 (2%)
Metastatic ovarian cancer	110 (2%)
Mucinous borderline tumour of endocervical type	6 (0.1%)
Mucinous borderline tumour of intestinal type	39 (1%)
Mucinous cystadenoma/mucinous cystadenofibroma	281 (6%)
Primary ovarian cancer	598 (12%)
Serous cystadenoma/serous cystadenofibroma	791 (16%)
Simple cyst/para-ovarian or salpingeal cyst	628 (13%)

© 2022 Timmerman S et al. *JAMA Oncology*.

Teratoma	532 (11%)
Not possible	164 (3%)

* Menopausal status at recruitment. If menopausal status was uncertain (e.g. because of hysterectomy), we classified patients aged 50 years or older as postmenopausal.

†Only for tumors with a solid component (n=1734 or 35%)

© 2022 Timmerman S et al. *JAMA Oncology*.

eTable 2. Outcome Determination by O-RADS Category

O-RADS	Overall, N (column %)	Immediate surgery[a], N (row %)	Delayed surgery[b], N (row %)	Spontaneous resolution, N (row %)	Subjective assessment up to 1 year, N (row %)	Uncertain (hence imputed), N (row %)
**O-RADS lexicon**						
O-RADS 2 (<1%)	2196 (44.8%)	727 (33.1%)	306 (13.9%)	329 (15.0%)	591 (26.9%)	243 (11.1%)
O-RADS 3 (1-<10%)	857 (17.5%)	348 (40.6%)	124 (14.5%)	93 (10.9%)	203 (23.7%)	89 (10.4%)
O-RADS 4 (10-<50%)	904 (18.4%)	584 (64.6%)	93 (10.3%)	28 (3.1%)	102 (11.3%)	97 (10.7%)
O-RADS 5 (≥50%)	939 (19.1%)	825 (87.9%)	27 (2.9%)	13 (1.4%)	19 (2.0%)	55 (5.9%)
Unclassified*	9 (0.2%)	5 (55.6%)	0 (0.0%)	2 (22.2%)	0 (0.0%)	2 (22.2%)
**IOTA two-step strategy**						
<1%	1984 (40.4%)	646 (32.6%)	278 (14.0%)	298 (15.0%)	545 (27.5%)	217 (10.9%)
1-<10%	1304 (26.6%)	542 (41.6%)	181 (13.9%)	134 (10.3%)	312 (23.9%)	135 (10.4%)
10-<50%	690 (14.1%)	480 (69.6%)	62 (9.0%)	25 (3.6%)	48 (7.0%)	75 (10.9%)
≥50%	927 (18.9%)	821 (88.6%)	29 (3.1%)	8 (0.9%)	10 (1.1%)	59 (6.4%)

[a] Operated within 120 days after the first scan without follow-up scan

© 2022 Timmerman S et al. *JAMA Oncology*.

[b] Operated after 120 days or after at least one follow-up scan

* It was not possible to classify 9 patients using the O-RADS lexicon because the tumor type was “unclassifiable“ in the International Ovarian Tumor Analysis database, and there was no ascites or metastasis

© 2022 Timmerman S et al. *JAMA Oncology*.

eTable 3. Observed Number and Percentage of Different Tumor Types in Each Ovarian-Adnexal Reporting and Data System (O-RADS) Risk Group When Using the O-RADS Lexicon and When Using the International Ovarian Tumor Analysis (IOTA) 2-Step Strategy to Estimate the Malignancy Risk (Pooled Analysis). The dataset with multiply imputed outcomes is used (n = 4905), which explains why frequencies are presented with decimals.

**O-RADS group, malignancy risk**	**Overall, N (column %)**	**Benign, N (row %)**	**Borderline, N (row %)**	**Stage I invasive, N (row %)**	**Stage II – IV invasive, N (row %)**	**Secondary metastatic, N (row %)**	**% malignant within group (95% CI)**
**Ultrasound features based on the O-RADS lexicon**							
O-RADS 2 (<1%)	2196 (44.8%)	2172.5 (98.9)	9.4 (0.4)	5.7 (0.3)	4.7 (0.2)	3.7 (0.2)	1.1 (0.7-1.6)
O-RADS 3 (1-<10%)	857 (17.5%)	823.4 (96.1)	16.3 (1.9)	6.4 (0.7)	5.5 (0.6)	5.4 (0.6)	3.9 (2.8-5.5)
O-RADS 4 (10-<50%)	904 (18.4%)	657.6 (72.7)	109.7 (12.1)	51.0 (5.6)	61.9 (6.8)	23.8 (2.6)	27.3 (24.4-30.3)
O-RADS 5 (≥50%)	939 (19.1%)	206.8 (22.0)	86.5 (9.2)	125.7 (13.4)	407.2 (43.4)	112.7 (12.0)	78.0 (75.2-80.5)
**IOTA two-step strategy**							
<1%	1984 (40.4 %)	1965.8 (99.1)	6.3 (0.3)	2.5 (0.1)	4.7 (0.2)	4.7 (0.2)	0.9 (0.6-1.5)
1-<10%	1304 (26.6 %)	1246.4 (95.6)	32.1 (2.5)	7.9 (0.6)	8.9 (0.7)	8.7 (0.7)	4.4 (3.4-5.7)

© 2022 Timmerman S et al. *JAMA Oncology*.

10-<50%	690<br>(14.1 %)	484.3<br>(70.2)	105.1<br>(15.2)	37.0<br>(5.4)	39.3<br>(5.7)	24.2<br>(3.5)	29.8<br>(26.4-33.4)
≥50%	927<br>(18.9 %)	171.5<br>(18.5)	78.6<br>(8.5)	141.4<br>(15.3)	427.4<br>(46.1)	108.1<br>(11.7)	81.5<br>(78.8-83.9)

ADNEX: Assessment of Different NEoplasias in the adnexa

* In the main manuscript, the frequencies and percentages were rounded to avoid confusion. These decimals are the result of using multiple imputation.

© 2022 Timmerman S et al. *JAMA Oncology*.

eTable 4. Observed Number and Percentage of Malignant Tumors in Each Ovarian-Adnexal Reporting and Data System (O-RADS) Subgroup Category (Pooled Analysis). The dataset with multiply imputed outcomes is used (n = 4905), which explains why some frequencies have decimals.

	Overall<br>N (column %)	Benign<br>N (row %)	Malignant<br>N (row %)
**O-RADS 2 (malignancy risk <1%)**	**2196 (44.8%)**	**2172.5 (98.9%)**	**23.5 (1.1%)**
– 2a: Simple cyst	692 (14.1%)	689.2 (99.6%)	2.8 (0.4%)
∗ 2a1: ≤ 3 cm	142 (2.9%)	140.89 (99.2%)	1.11 (0.8%)
∗ 2a2: > 3 to 5 cm	294 (6.0%)	292.7 (99.6%)	1.3 (0.4%)
∗ 2a3: > 5cm but < 10 cm	256 (5.2%)	255.61 (99.9%)	0.39 (0.2%)
– 2b: Classic benign lesions	1362 (27.8%)	1347.47 (98.8%)	14.53 (1.1%)
∗ 2b1: Typical hemorrhagic cyst < 10 cm	68 (1.4%)	67.94 (99.9%)	0.06 (0.1%)
∗ 2b2: Typical dermoid cyst < 10 cm	464 (9.5%)	460.65 (99.3%)	3.35 (0.7%)
∗ 2b3 : Typical endometrioma < 10 cm	694 (14.1%)	687.46 (99.1%)	6.54 (0.9%)
Peritoneal inclusion cyst	23 (0.5%)	21.9 (95.2%)	1.1 (4.8%)
Hydrosalpinx	113 (2.3%)	109.52 (96.9%)	3.48 (3.1%)
– 2c Non-simple unilocular cyst, smooth inner margin	142 (2.9%)	135.83 (95.7%)	6.17 (4.4%)
∗ 2c1 : ≤ 3 cm	22 (0.4%)	21.94 (99.7%)	0.06 (0.3%)
∗ 2c2 : > 3 but < 10 cm	120 (2.4%)	113.89 (94.9%)	6.11 (5.1%)
**O-RADS 3 (malignancy risk 1-<10%)**	**857 (17.5%)**	**823.37 (96.1%)**	**33.63 (3.9%)**
– 3a: Unilocular cyst ≥ 10 cm (simple or non-simple)	88 (1.8%)	82.74 (94.0%)	5.26 (6.0%)
– 3b: Typical dermoid cysts, endometrioma, hemorrhagic cysts ≥ 10 cm	79 (1.6%)	72.9 (92.3%)	6.1 (7.7%)
∗ 3b1: Typical hemorrhagic cyst ≥ 10 cm	0 (0.0%)	0 (0.0%)	0 (0.0%)
∗ 3b2: Typical dermoid cyst ≥ 10 cm	47 (1.0%)	43.96 (93.5%)	3.04 (6.5%)
∗ 3b3 : Typical endometrioma ≥ 10 cm	32 (0.7%)	28.94 (90.4%)	3.06 (9.6%)
– 3c: Unilocular cyst, any size with irregular wall < 3 mm height	123 (2.5%)	110.19 (89.6%)	12.81 (10.4%)
– 3d: Multilocular cyst <10 cm, smooth inner wall, Color score = 1-3	470 (9.6%)	462.04 (98.3%)	7.96 (1.7%)
– 3e: Solid smooth, any size, Color score = 1	97 (2.0%)	95.5 (98.5%)	1.5 (1.5%)
**O-RADS 4 (malignancy risk 10-<50%)**	**904 (18.4%)**	**657.6 (72.7%)**	**246.4 (27.3%)**
– 4a: Multilocular cyst, no solid component	279 (5.7%)	224.6 (80.5%)	54.4 (19.5%)

© 2022 Timmerman S et al. *JAMA Oncology*.

∗ 4a1: ≥ 10 cm, smooth inner wall, Color score = 1-3	142 (2.9%)	115.7 (81.5%)	26.3 (18.5%)
∗ 4a2: Any size, smooth inner wall, Color score = 4	4 (0.1%)	1.86 (46.5%)	2.14 (53.5%)
∗ 4a3: Any size, irregular inner wall and/or irregular septation, Color score = any	133 (2.7%)	107.04 (80.5%)	25.96 (19.5%)
– 4b: Unilocular cyst with solid component, any size, 0-3 papillary projections, Color score = any	267 (5.4%)	162.61 (60.9%)	104.39 (39.1%)
– 4c: Multilocular cyst with solid component, any size, Color score = 1-2	183 (3.7%)	133.87 (73.2%)	49.13 (26.8%)
– 4d: Solid, Smooth, any size, Color score = 2-3	175 (3.6%)	136.52 (78.0%)	38.48 (22.0%)
**O-RADS 5 (malignancy risk ≥50%)**	**939 (19.1%)**	**206.83 (22.0%)**	**732.17 (78.0%)**
– 5a: Unilocular cyst, any size, ≥ 4 papillary projections, Color score = any	54 (1.1%)	15.02 (27.8%)	38.98 (72.2%)
– 5b: Multilocular cyst with solid component, any size, Color score = 3-4	304 (6.2%)	110.4 (36.3%)	193.6 (63.7%)
– 5c: Solid smooth, any size, Color score = 4	57 (1.2%)	18.73 (32.9%)	38.27 (67.1%)
– 5d: Solid irregular, any size, Color score = any	94 (1.9%)	30.06 (32.0%)	63.94 (68.0%)
– 5e: Ascites and/or peritoneal nodules	430 (8.8%)	32.62 (7.6%)	397.38 (92.4%)
**Uncertain allocation**	9 (0.2%)	7.71 (85.7%)	1.29 (14.3%)

It was not possible to classify 9 patients using the O-RADS lexicon because the tumor type was “unclassifiable“ in the International Ovarian Tumor Analysis database, and there was no ascites or metastasis

© 2022 Timmerman S et al. *JAMA Oncology*.

eTable 5. Observed Prevalence of Malignancy per O-RADS Group Depending on Menopausal Status, Type of Center, or the Actual Management When Using the Ovarian-Adnexal Reporting and Data System (O-RADS) Lexicon and the International Ovarian Tumor Analysis (IOTA) 2-Step Strategy to Estimate the Risk of Malignancy (Pooled Analysis)

	**O-RADS lexicon**		**IOTA two-step strategy**	
**Premenopausal patients (n=2754; 14% malignant)**	**N**	**Malignancy rate (95% CI)**	**N**	**Malignancy rate (95% CI)**
O-RADS 2 (<1%)	1604	0.8 (0.4-1.5)	1413	0.6 (0.2-1.2)
O-RADS 3 (1-<10%)	416	3.9 (2.3-6.3)	706	4.5 (3.1-6.3)
O-RADS 4 (10-<50%)	378	30.3 (25.8-35.2)	335	34.8 (29.8-40.2)
O-RADS 5 (≥50%)	348	71.2 (66.1-75.8)	300	78.6 (73.5-83.0)
**Postmenopausal patients (n=2151; 30% malignant)**	**N**	**Malignancy rate (95% CI)**	**N**	**Malignancy rate (95% CI)**
O-RADS 2 (<1%)	592	1.8 (0.9-3.3)	571	1.8 (0.9-3.4)
O-RADS 3 (1-<10%)	441	4.0 (2.5-6.4)	598	4.4 (3.0-6.4)
O-RADS 4 (10-<50%)	526	25.1 (21.4-29.1)	355	25.1 (20.7-30.0)
O-RADS 5 (≥50%)	591	82.0 (78.6-84.9)	627	82.9 (79.7-85.7)
**Patients examined in an oncology center (n=3094; 28% malignant)**	**N**	**Malignancy rate (95% CI)**	**N**	**Malignancy rate (95% CI)**
O-RADS 2 (<1%)	1275	1.4 (0.8-2.2)	1094	1.2 (0.6-2.1)
O-RADS 3 (1-<10%)	478	5.0 (3.4-7.5)	725	4.6 (3.3-6.5)
O-RADS 4 (10-<50%)	601	32.0 (28.3-35.9)	463	28.7 (24.6-33.1)
O-RADS 5 (≥50%)	731	84.4 (81.5-86.9)	812	82.8 (80.0-85.3)

© 2022 Timmerman S et al. *JAMA Oncology*.

**Patients examined in a non-oncology center (n=1811; 10% malignant)**	**N**	**Malignancy rate (95% CI)**	**N**	**Malignancy rate (95% CI)**
O-RADS 2 (<1%)	921	0.7 (0.3-1.5)	890	0.6 (0.2-1.5)
O-RADS 3 (1-<10%)	379	2.5 (1.3-4.7)	579	4.2 (2.8-6.2)
O-RADS 4 (10-<50%)	303	17.8 (13.8-22.7)	227	32.1 (26.2-38.7)
O-RADS 5 (≥50%)	208	55.5 (48.6-62.2)	115	72.0 (62.9, 79.6)
**Immediately operated* (n=2489; 38% malignant)**	**N**	**Malignancy rate (95% CI)**	**N**	**Malignancy rate (95% CI)**
O-RADS 2 (<1%)	727	2.3 (1.5-3.7)	646	2.0 (1.2-3.4)
O-RADS 3 (1-<10%)	348	8.0 (5.6-11.4)	542	8.5 (6.4-11.1)
O-RADS 4 (10-<50%)	584	36.1 (32.3-40.1)	480	37.5 (33.3-41.9)
O-RADS 5 (≥50%)	825	83.4 (80.7-85.8)	821	86.0 (83.4-88.2)
**At least one follow-up scan (n=1958; 2% malignant)**	**N**	**Malignancy rate (95% CI)**	**N**	**Malignancy rate (95% CI)**
O-RADS 2 (<1%)	1223	0.3 (0.1-1.0)	1117	0.2 (0-1.0)
O-RADS 3 (1-<10%)	433	0.9 (0.3-2.6)	644	1.1 (0.4-2.5)
O-RADS 4 (10-<50%)	247	9.3 (5.9-14.1)	153	12.6 (7.8-19.6)
O-RADS 5 (≥50%)	53	9.8 (3.7-22.8)	44	15.9 (7.2-31.3)

* Operated within 120 days after the first scan without follow-up scan

Unclassified patients based on O-RADS lexicon: 8 for premenopausal (3.6% malignancy rate); 1 for postmenopausal (100% malignancy rate); 9 for patients examined in an oncology center (14.3% malignancy rate); 0 for patients examined in a non-oncology center, 5 for immediately operated (20% malignancy rate) and 2 for patients with at least one follow-up scan (0% malignancy rate).

© 2022 Timmerman S et al. *JAMA Oncology*.

eTable 6. Sensitivity and Specificity With Regard to Malignancy of the Ovarian-Adnexal Reporting and Data System (O-RADS) Lexicon and International Ovarian Tumor Analysis (IOTA) 2-Step Strategy Depending on Menopausal Status, Type of Center, and Actual Management (Meta-analysis)

Cut-off for malignancy for O-RADS lexicon and for percentage risk of malignancy	**O-RADS lexicon**	**IOTA two-step strategy**	**O-RADS lexicon**	**IOTA two-step strategy**
**Premenopausal patients (n=2754)**	**Sensitivity (95% CI)**		**Specificity (95% CI)**	
O-RADS 3; 1%	0.95 (0.90 - 0.97)	0.95 (0.88 - 0.98)	0.68 (0.62 - 0.73)	0.60 (0.54 - 0.65)
O-RADS 4; 10%	0.89 (0.82 - 0.94)	0.88 (0.79 - 0.93)	0.85 (0.81 - 0.88)	0.89 (0.85 - 0.92)
O-RADS 5; 50%	0.59 (0.50 - 0.68)	0.54 (0.43 - 0.64)	0.96 (0.94 - 0.97)	0.98 (0.96 - 0.99)
**Postmenopausal patients (n=2151)**	**Sensitivity (95% CI)**		**Specificity (95% CI)**	
O-RADS 3; 1%	0.97 (0.95 - 0.99)	0.97 (0.94 - 0.98)	0.41 (0.34 - 0.48)	0.40 (0.33 - 0.46)
O-RADS 4; 10%	0.94 (0.89 - 0.97)	0.93 (0.86 - 0.97)	0.70 (0.62 - 0.76)	0.77 (0.70 - 0.82)
O-RADS 5; 50%	0.71 (0.64 - 0.78)	0.77 (0.66 - 0.86)	0.95 (0.91 - 0.97)	0.93 (0.90 - 0.96)
**Patients examined in an oncology center (n=3094)**	**Sensitivity (95% CI)**		**Specificity (95% CI)**	
O-RADS 3; 1%	0.97 (0.94 - 0.99)	0.97 (0.95 - 0.99)	0.58 (0.46 – 0.70)	0.50 (0.40 - 0.60)
O-RADS 4; 10%	0.94 (0.87 - 0.97)	0.94 (0.88 - 0.97)	0.79 (0.70 - 0.87)	0.81 (0.75 - 0.86)
O-RADS ; 50%	0.70 (0.61 - 0.77)	0.78 (0.74 - 0.82)	0.96 (0.93 - 0.97)	0.94 (0.92 - 0.96)
**Patients examined in a non-oncology center (n=1811)**	**Sensitivity (95% CI)**		**Specificity (95% CI)**	
O-RADS 3; 1%	0.96 (0.90 - 0.98)	0.95 (0.81 - 0.99)	0.57 (0.54 - 0.60)	0.56 (0.52 - 0.60)
O-RADS 4; 10%	0.88 (0.79 - 0.93)	0.84 (0.67 - 0.93)	0.81 (0.76 - 0.85)	0.89 (0.86 - 0.92)
O-RADS 5; 50%	0.57 (0.44 - 0.69)	0.43 (0.34 - 0.52)	0.97 (0.93 - 0.99)	0.98 (0.97 - 0.99)
**Immediately operated* (n=2489)**	**Sensitivity (95% CI)**		**Specificity (95% CI)**	
O-RADS 3; 1%	0.98 (0.96 - 0.99)	0.97 (0.94 - 0.98)	0.45 (0.38 - 0.53)	0.41 (0.36 - 0.47)
O-RADS 4; 10%	0.94 (0.90 - 0.96)	0.92 (0.86 - 0.96)	0.68 (0.60 - 0.74)	0.74 (0.67 - 0.80)
O-RADS 5; 50%	0.69 (0.62 - 0.75)	0.69 (0.58 - 0.79)	0.93 (0.89 - 0.95)	0.93 (0.89 - 0.95)
**At least one follow-up scan (n=1958)**	**Sensitivity (95% CI)**		**Specificity (95% CI)**	
O-RADS 3; 1%	0.77 (0.45 - 0.93)	0.81 (0.43 - 0.96)	0.66 (0.58 - 0.74)	0.60 (0.52 - 0.67)
O-RADS 4; 10%	0.59 (0.27 - 0.84)	0.60 (0.30 - 0.84)	0.88 (0.83 - 0.91)	0.91 (0.88 - 0.93)
O-RADS 5; 50%	0.24 (0.09 - 0.52)	0.20 (0.07 - 0.45)	0.98 (0.96 - 0.99)	0.97 (0.96 - 0.98)

Value at cutoff or higher classifies the mass as malignant

* Operated within 120 days after the first scan without follow-up scan

© 2022 Timmerman S et al. *JAMA Oncology*.

eTable 7. Observed Prevalence of Malignancy in Ovarian-Adnexal Reporting and Data System (O-RADS) Groups 3a, 3d, 3e, 4d, and 5c When Number of Cyst Locules (O-RADS 3d), Echogenicity of Cyst Fluid (O-RADS 3a, 3d), and Shadowing (O-RADS 3e, 4d, and 5c) Are Taken Into Account (Pooled Analysis)

The dataset with multiply imputed outcomes is used (n = 4905), which explains why some frequencies have decimals.

	Overall N (column %)	Malignant after multiple imputation N (row %)
**O-RADS 2**	**2196 (44.8%)**	**23.5 (1.1%)**
…		
**O-RADS 3**	**857 (17.5%)**	**33.63 (3.9%)**
– 3a: Unilocular cyst ≥ 10 cm	88 (1.8%)	5.26 (6.0%)
Anechoic content	54 (1.1%)	0.14 (0.3%)
Other content	34 (0.7%)	5.12 (15.1%)
…		
– 3d Multilocular cyst <10 cm, smooth inner wall, Color score = 1-3	470 (9.6%)	7.96 (1.7%)
Bilocular	183 (3.7%)	1.37 (0.7%)
Anechoic content	159 (3.2%)	0.14 (0.1%)
Other content	24 (0.5%)	1.23 (5.1%)
> 2 locules	287 (5.9%)	6.59 (2.3%)
– 3e Solid smooth, any size, Color score=1	97 (2.0%)	1.5 (1.5%)
With acoustic shadows	64 (1.3%)	0.08 (0.1%)
Without acoustic shadows	33 (0.7%)	1.42 (4.3%)
**O-RADS 4**	**904 (18.4%)**	**246.4 (27.3%)**
…		
– 4d Solid, Smooth, any size, Color score= 2-3	175 (3.6%)	38.48 (22.0%)
With acoustic shadows	81 (1.7%)	3.23 (4.0%)
Without acoustic shadows	94 (1.9%)	35.25 (37.5%)
**O-RADS 5**	**939 (19.1%)**	**732.17 (78.0%)**
…		
– 5c Solid smooth, any size, Color score= 4	57 (1.2%)	38.27 (67.1%)
With acoustic shadows	1 (0.0%)	0 (0.0%)
Without acoustic shadows	56 (1.1%)	38.27 (68.3%)
…		
**Uncertain allocation**	9 (0.2%)	1.29 (14.3%)

© 2022 Timmerman S et al. *JAMA Oncology*.

eAppendix. Search Strategy to Find Publications on Validation of Ovarian Adnexal Reporting and Data System (O-RADS)

We searched PubMed (National Library of Medicine), Embase (Elsevier, via Embase.com) and CINAHL Complete (EbscoHost). Terms for O-RADS and Ovarian Adnexal Reporting and Data System were used to conduct a broad search. No publication date restriction or language restriction were used. No filters were applied. The search strategy was developed in collaboration with an information specialist.

1. PubMed (National Library of Medicine)
Date of search 05-05-2022
No language or publication date restrictions were used

[Search string below in All Fields]

O-RADS OR ovarian adnexal reporting data system

87 records

2. Embase (Elsevier, via Embase.com)
Date of search 05-05-2022
No language or publication date restrictions were used

[Search string below in All Fields]

#1 ('o rads' OR ovarian) AND adnexal AND reporting AND data AND system
61 records

#2 AND [embase]/lim NOT ([embase]/lim AND [medline]/lim)

20 records

#3 #2 NOT 'conference abstract':it

17 Records

3. CINAHL Complete (EbscoHost)

Date of search: 05-05-2022
No language or publication date restrictions were used

[Search string below in All Fields]

O-RADS OR ovarian adnexal reporting data system

34 records

Filter Academic Journals

29 records

In total 133 records from database searches. Of these, 11 described validation of O-RADS.

In addition, one references was found by forward snowballing of Andreotti et al. (doi:10.1148/radiol.2019191150), one in Google Scholar (published 18-05-2022) and one in Pubmed (published 01-06-2022).

© 2022 Timmerman S et al. *JAMA Oncology*.

eTable 8. Summary of Studies Validating the Ovarian Adnexal Reporting and Data System (O-RADS)

	The current study	Chen et al. 2022[1]	Lai et al. 2021[2]	Hiett et al. 2022[3]	Hack et al. 2022[4]	Wang et al. 2022[5]	Xie et al. 2022[6]	Guo et al. 2022[7]	Basha et al. 2021[8]	Cao et al. 2021[9]	Chen et al. 2022[10]	Jha et al. 2022[11]	Solis et al. 2021[12]	Pi et al. 2021[13]	Ahmed et al. 2021[14]
Sample size	4905 patients	322 patients	734 patients	150 patients	227 patients; 262 lesions	431 patients	453 patients	575 patients 592 lesions	609 patients 647 lesions	1035 patients; 1054 lesions	85 patients (validation set)	913 patients; 1014 lesions.	73 patients	50 patients	50 patients with 'suspicious ovarian mass lesions'
Setting	Multicenter study: 17 centers in 7 countries, both oncology centers and others	Single tertiary referral oncology center, Taiwan	Single center university hospital, China	Single tertiary referral oncology center, USA	Single tertiary referral oncology center, Canada	Single research hospital, China	Single tertiary referral oncology center, China	Single center unspecified, China	Multicenter: 3 unspecified centers, Egypt	Multicenter: 1 unspecified referral center and 1 cancer center, China	Single center university hospital, China	Multicenter: 6 academic radiology departments , USA	Single tertiary referral oncology center, Mexico	Single center unspecified, USA	Single center university hospital, Egypt
Method of applying the O-RADS lexicon	Retrospective analysis of data in IOTA 5 database. Translation of IOTA terminology to O-RADS lexicon. IOTA two-step strategy (ADNEX without CA125) was validated retrospectively	Retrospective review of images by 2 experienced gynecologists. ADNEX +/- CA125 was validated retrospectively. Consensus of the reviewers was used.	Retrospective review of images by 2 junior resident sonologists (5 years experience), their consensus was used. ADNEX with CA125 was validated retrospectively	Retrospective review of images and recorded ultrasound variables by 2 experienced sonologists. Consensus used? ADNEX without CA125 was prospectively used clinically	Retrospective review of images and video clips by 2 subspecialist radiologists, their consensus was used (one discrepancy) ADNEX +/-CA125 was validated retrospectively	Retrospective analysis but unclear of what and by whom: images? ultrasound reports?	Retrospective review of images by 2 radiologists (> 5 years of experience), their consensus was used	Retrospective review of images by 2 subspecialist radiologists (group I) or by 2 junior doctors (group II), the consensus of the two reviewers was used	Retrospective review of images and ultrasound reports by 5 consultant radiologists, their consensus was used	Retrospective review of images by 2 first year residents in radiology, their consensus was used	Retrospective review of images by 1 expert ultrasound examiner	Retrospective review of images by 8 experienced radiologists. Consensus between 2 readers was used	Retrospective review of images by 1 radiologist	Retrospective review of images by 3 radiologists; results reported for each of the three reviewers	Prospective study Unclear who used O-RADS (radiologist ? gynecologist? level of experience? one or more examiners?)
Reference standard	Histology or FU	Histology	Histology	Histology	Histology or FU with tumor growth >20% over 2 years defined as malignant tumor	Histology	Histology	Histology	Histology (n=409) or FU every 3 months for at least 2 years	Histology	Histology	Histology or FU (pathologic diagnosis or benign defined as resolution or decrease in size by 10% on FU imaging; classic lesion on CT or MRI; stability on imaging for > 2 years or documented normal pelvic examination > 2 years after initial pelvic ultrasound.	Histology	Unclear: Consensus of three radiologists, so based on image analysis? No histology	Histology (n=39) or FU (6-12 months before assigning a benign clinical diagnosis)
Malignancy rate (%) in															
O-RADS 2	1.1	0	1.36	0	0	1.3	5.13	2.0 (I); 2.8 (II)	0.4	0.45	Not reported	0.5	Not reported	Not reported	Not reported
O-RADS 3	3.9	3.0	4.37	0	3	11.8	18.64	8.9 (I) 13.9 (II)	2.8	1.10	Not reported	4.5	Not reported	Not reported	15.38
O-RADS 4	27.3	37.7	58.06	21.21	35	55.8	61.19	52.3 (I) 42.9 (II)	30.6	34.46	Not reported	11.6	Not reported	Not reported	78.95
O-RADS 5	78.0	78.9	94.52	78.79	78	90.8	94.50	86.7 (I) 81.5 (II)	95.3	89.57	Not reported	65.6	Not reported	Not reported	94.44

© 2022 Timmerman S et al. *JAMA Oncology*.

Sensitivity (%) O-RADS 4-5 (10% risk)	92	96.6	88	100	99	preMP: 92.2<br>postMP: 94.8	94.4	91.0 (I)<br>84.8 (II)	96.6	98.7	92	90.6	Not reported	91.7; 91.7; 91.7	94.12
Specificity (%) ORADS 4-5 (10% risk)	80	78.5	94	46	70	preMP: 91.8<br>postMP: 83.9	63.6	81.9 (I)<br>81.9 (II)	92.8	83.2	89	81.9	Not reported	92.1; 91.2; 97.4	68.75
Sensitivity (%) ADNEX (10% risk)	91	91.4 (+CA125)<br>91.4 (-CA125)	95	97.5	NA	NA	NA	NA	NA	NA	NA	NA	NA	NA	NA
Specificity (%) ADNEX (10% risk)	85	78.9 (+CA125)<br>79.5 (-CA125)	86	63.6	NA	NA	NA	NA	NA	NA	NA	NA	NA	NA	NA
Comments		Compare O-RADS lexicon with ADNEX in patients with pre-operative CA125 (281 out of 322)	Compare sensitivity and specificity of G-RADS, O-RADS and ADNEX<br>Results for prevalence of malignancy in the O-RADS risk groups are not presented by the authors but are derived by us from their Table 3	Compare sensitivity, specificity, and AUC of O-RADS, ADNEX, Simple Rules, and SRRisk model.<br>AUC ADNEX 0.937<br>AUC SRRisk 0.941<br>AUC O-RADS and Simple Rules not reported.<br>Information on IOTA variables was collected prospectively	Partly inappropriate reference standard (growth >20% over 2 years defined as malignant tumor).<br>2198/ 2801 (78%) patients excluded because of inadequate FU.<br>Report mean ADNEX risk per O-RADS group.<br>AUC O-RADS 0.91<br>AUC ADNEX 0.95	Separate analysis for preMP and postMP patients<br>AUC preMP: 0.95<br>AUC postMP: 0.91	Report sensitivity, specificity and AUC of O-RADS, Simple Rules, and CA125<br>AUC O-RADS: 0.804<br>AUC Simple Rules: 0.831<br>AUC CA 125: 0.812	Report sensitivity, specificity, and AUC of ORADS; RMI4; IOTA LR2 and IOTA simple rules	Compare sensitivity, specificity and AUC of O-RADS, GIRADS, and IOTA Simple Rules<br>AUC O-RADS 0.98<br>AUC GIRADS 0.97<br>AUC Simple Rules 0.94	No validation of other methods	Compare sensitivity, specificity, and AUC of Deep Learning, O-RADS, and expert subjective assessment.<br>AUC O-RADS 0.92;<br>AUC $DL_{decision}$ 0.90;<br>AUC $DL_{feature}$ 0.93;<br>AUC subjective assessment 0.97	No validation of other methods.	For O-RADS threshold 2 (risk ≥1%) sensitivity 52%, specificity 93.75%<br>No validation of other methods	Inappropriate reference standard<br>AUC also reported: 0.942, 0.975, 0.983<br>No validation of other methods	Included only O-RADS 3-5<br>Malignancy rate per O-RADS group not reported but derived from their Table 5<br>Not in Pub Med<br>No validation of other methods

ADNEX, Assessment of Different NEoplasias in the adnexa-model; AUC, Area under the receiver operating characteristics curve; DL, deep learning. FU, Follow-up; GIRADS, Gynecologic Imaging-Reporting and Data System; IOTA, International Ovarian Tumor Analysis group; LR2, logistic regression model 2; MP, menopause or menopausal; NA, ADNEX not validated; RMI, Risk of Malignancy Index; SRRisk, Simple Rules Risk calculation; CT, Computed tomography; MRI, magnetic resonance imaging.

© 2022 Timmerman S et al. *JAMA Oncology*.

eReferences.

1. Chen, GY., Hsu, TF., Chan, IS. et al. Comparison of the O-RADS and ADNEX models regarding malignancy rate and validity in evaluating adnexal lesions. Eur Radiol (2022). https://doi.org/10.1007/s00330-022-08803-6
2. Lai HW, Lyu GR, Kang Z, Li LY, Zhang Y, Huang YJ. Comparison of O-RADS, GI-RADS, and ADNEX for Diagnosis of Adnexal Masses: An External Validation Study Conducted by Junior Sonologists [published online ahead of print, 2021 Sep 21]. J Ultrasound Med. 2021;10.1002/jum.15834. doi:10.1002/jum.15834
3. Hiett AK, Sonek JD, Guy M, Reid TJ. Performance of IOTA Simple Rules, Simple Rules risk assessment, ADNEX model and O-RADS in differentiating between benign and malignant adnexal lesions in North American women [published online ahead of print, 2021 Sep 17]. Ultrasound Obstet Gynecol. 2021;10.1002/uog.24777. doi:10.1002/uog.24777
4. Hack K, Gandhi N, Bouchard-Fortier G, et al. External Validation of O-RADS US Risk Stratification and Management System [published online ahead of print, 2022 Apr 19]. Radiology. 2022;211868. doi:10.1148/radiol.211868
5. Wang R, Li X, Li S, et al. Clinical value of O-RADS combined with serum CA125 and HE4 for the diagnosis of ovarian tumours [published online ahead of print, 2022 Mar 15]. Acta Radiol. 2022;2841851221087376. doi:10.1177/02841851221087376
6. Xie WT, Wang YQ, Xiang ZS, et al. Efficacy of IOTA simple rules, O-RADS, and CA125 to distinguish benign and malignant adnexal masses. J Ovarian Res. 2022;15(1):15. Published 2022 Jan 23. doi:10.1186/s13048-022-00947-9
7. Guo Y, Zhao B, Zhou S, et al. A comparison of the diagnostic performance of the O-RADS, RMI4, IOTA LR2, and IOTA SR systems by senior and junior doctors [published online ahead of print, 2022 Jan 31]. Ultrasonography. 2022;10.14366/usg.21237. doi:10.14366/usg.21237
8. Basha MAA, Metwally MI, Gamil SA, et al. Comparison of O-RADS, GI-RADS, and IOTA simple rules regarding malignancy rate, validity, and reliability for diagnosis of adnexal masses. Eur Radiol. 2021;31(2):674-684. doi:10.1007/s00330-020-07143-7
9. Cao L, Wei M, Liu Y, et al. Validation of American College of Radiology Ovarian-Adnexal Reporting and Data System Ultrasound (O-RADS US): Analysis on 1054 adnexal masses. Gynecol Oncol. 2021;162(1):107-112. doi:10.1016/j.ygyno.2021.04.031
10. Chen, Hui, et al. "Deep Learning Prediction of Ovarian Malignancy at US Compared with O-RADS and Expert Assessment." Radiology (2022): 211367. https://doi.org/10.1148/radiol.211367
11. Jha P, Gupta A, Baran TM, et al. Diagnostic Performance of the Ovarian-Adnexal Reporting and Data System (O-RADS) Ultrasound Risk Score in Women in the United States. JAMA Netw Open. 2022;5(6):e2216370. Published 2022 Jun 1. doi:10.1001/jamanetworkopen.2022.16370
12. Solis Cano DG, Cervantes Flores HA, De Los Santos Farrera O, Guzman Martinez NB, Soria Céspedes D. Sensitivity and Specificity of Ultrasonography Using Ovarian-Adnexal Reporting and Data System Classification Versus Pathology Findings for Ovarian Cancer. Cureus. 2021;13(9):e17646. Published 2021 Sep 1. doi:10.7759/cureus.17646
13. Pi Y, Wilson MP, Katlariwala P, et al. Diagnostic accuracy and inter-observer reliability of the O-RADS scoring system among staff radiologists in a North American academic clinical setting. Abdom Radiol (NY). 2021;46(10):4967-4973. doi:10.1007/s00261-021-03193-7.
14. Ahmed, H. E. K. (2021). The usefulness of the ultrasound diagnosis of suspicious ovarian masses based on the O-RADS classification system. Al-Azhar International Medical Journal.

© 2022 Timmerman S et al. *JAMA Oncology*.
